# An uncommon onset for tricuspid endocarditis

**DOI:** 10.1186/1749-8090-10-S1-A129

**Published:** 2015-12-16

**Authors:** Marta Matamala, Elisa Ochoa, Neslim Gálvez, Javier Fañanás, Jose Maria Vallejo, Manuel Vázquez, Fernando Sorribas, Carlos Ballester

**Affiliations:** 1 Servicio de Cirugía Cardiovascular, Hospital Universitario Miguel Servet, Zaragoza, Spain

## Background/Introduction

Tricuspid endocarditis itself is a less common cause of endocarditis. It is rarely seen in non-intravenous drug users, non-central venous catheter, non-pacemaker patients and patients without congenital cardiac disorder.

## Aims/Objectives

We describe the case of a young patient with tricuspid valve endocarditis who had an uncommon entry of bacteria.

## Method

The patient was referred to surgery because of her clinical course worsened with persistent fever, big vegetations and pulmonary embolism. After meticulous examination of the patient we found a scar under her breast. There she had an abscess two months ago that healed spontaneously.

## Results

Surgical indication was for pulmonary embolism and big vegetations. We did cleaning and debridement of the tricuspid valve, and took out three vegetations of approximately one centimeter each. Postoperative course had no incidences and finally she was discharged after completing antibiotic treatment.

## Discussion/Conclusion

Most of the cases of tricuspid endocarditis (nearly 80%) are found in intravenous drug users. This case has a very rare entry of bacteria. First, because it started nearly two months ago and second because the skin infection almost had disappeared when the patient was diagnosed of endocarditis.

There is no standardized surgical procedure for tricuspid endocarditis. In this case, as regurgitation was mild, we decided not to leave prosthetic material.

## Consent

Written informed consent was obtained from the patient for publication of this abstract and any accompanying images. A copy of the written consent is available for review by the Editor of this journal

